# Supplementary material for: Deep learning-based electrocardiographic screening for chronic kidney disease
Source: Commun Med (Lond). 2023 May 26;3:73. doi: 10.1038/s43856-023-00278-w (PMC10220039; doi:10.1038/s43856-023-00278-w)
Supplement: Supplementary file 1 — Supplementary Information [file 43856_2023_278_MOESM1_ESM.pdf]

Supplementary Information

Deep Learning Algorithm Training

Based on prior literature regarding lightweight deep learning model architecture design and neural architecture search[1,2], our deep learning model was designed to analyze 12-lead ECG waveform data starting with atrous convolutions followed by subsequent multi-channel 1D convolutions. The number of layers paralleled the design of EfficientNet[2], and to optimize model runtime and minimize model complexity, the number of layers were limited to less than 1/10th the size of previously described architectures.[3-5] After initial atrous layers, the model incorporated convolutional layers with an inverted residual structure where the input and output are bottleneck 9 layers with an intermediate expansion layer.[1] In each set of expansion layers with bottleneck layers preceding and succeeding, the number of input channels gradually increased to allow for integration of information across ECG leads. This model was previously used in prior work on ECG deep learning predicting other tasks[6] with code available online[7].

Model training was performed on a Linux (Ubuntu) computer with an 24 core AMD Threadripper 2960X and two RTX 2090s with a total of 48GB VRAM. The model was initialized with random weights and trained with a loss function of binary cross entropy for 100 epochs using an ADAM optimizer with an initial learning rate between 5e-3 and 1e-4. Early stopping was performed based on validation dataset's area under the receiver operating curve. The atrous convolution's dilation and step size was grid-searched by hyperparameter tuning for optimal AUC with all other hyperparameters held constant (Supplemental Figure 1). Local Interpretable Model-agnostic Explanations35 was used to identify and visualize relevant features in the ECG used for model decision making.

Disease	ICD9/10-code(s)
Any stage CKD	585.1+585.2+585.3+585.4+585.5+585.6
Mild stage CKD	585.1+585.2
Moderate-severe CKD	585.3+585.4+585.5
ESRD	585.6
Hypertension	401+I10+I11+I12+I13+I15
Diabetes Mellitus	250+E10+E11
Cardiovascular disease	410-414+430-438+I63+I64.9+I25+Z98.6+I70+I71+I73.9+I70.1
Heart failure	428+I25.5+I50+I11.0+I13.0+I13.2+I42.0+I42.5-I42.9+I43
Proteinuria	791.0
Anemia	280-285

Supplementary Table 1. List of ICD9/10-codes that were used in the present study. CKD=Chronic kidney disease. ESRD=End-stage renal disease.

Characteristic	Patients <60 years	Patients >60 years
Number of patients	41,657	56,055
Number of ECGs	80,011	138,370
**Demographics**		
Age, years	44.47 ±12.37	75.36 ±9.9
Female, n (%)	19,674 (47.2%)	27,305 (48.7%)
BMI, kg/m^2	27.96 ±16.63	26.05 ±17.73
Caucasian, n (%)	23,153 (55.6%)	36,837 (65.7%)
Black, n (%)	7,049 (16.9%)	6,792 (12.1%)
Asian, n (%)	2,387 (5.7%)	3,085 (5.5%)
Other/unknown race, n (%)	8,777 (21.1%)	9,010 (16.1%)
**Clinical characteristics, n (%)**		
Hypertension	6,260 (15.0%)	19,502 (34.8%)
Diabetes Mellitus	2,940 (7.1%)	8,521 (15.2%)
Cardiovascular disease	4,357 (10.5%)	9,899 (17.7%)
Heart Failure	2,899 (7.0%)	10,683 (19.1%)
Proteinuria	464 (1.1%)	458 (0.8%)
Anemia	4,514 (10.8%)	6,321 (11.3%)
**Chronic kidney disease, n (%)**		
Mild (Stage 1 -2)	333 (0.8%)	635 (1.1%)
Moderate (Stage 3-5)	982 (2.4%)	2,960 (5.3%)
ESRD	1,787 (4.3%)	1,308 (2.3%)
**eGFR, ml/min/1.73 m^2**		
<15	1,052 (2.5%)	1,368 (2.4%)
15-29	435 (1.0%)	2,108 (3.8%)
30-60	1,573 (3.8%)	8,944 (16.0%)
>60	16,078 (38.6%)	16,795 (30.0%)

Supplementary Table 2. Demographics and clinical characteristics according to the age group in the internal cohort. Continuous variables are presented as mean ± standard deviation. BMI=Body mass index, eGFR=estimated glomerular filtration rate, ESRD=End-stage renal disease.

Test task	AUC (95% CI)
eGFR<60 ml/min/1.73 m^2*	0.754 (0.737-0.771)
Albuminuria**	0.734 (0.723 - 0.745)
CKD among ambulatory patients	0.763 (0.74-785)
CKD among in-hospital patients	0.762 (0.752-0.772)
CKD among patients with K >5.5mmol/L***	0.741 (0.698 - 0.787)
CKD among patients with K <=5.5mmol/L***	0.758 (0.747 - 0.768)
White patients	0.764 (0.752 - 0.777)
Black patients	0.760 (0.738 - 0.779)
Asian patients	0.749 (0.715 - 0.782)
Unknown or unspecified race patients	0.769 (0.74 - 0.802)

Supplementary Table 3. 12-lead ECG based deep learning model's performance in additional tests in the internal cohort. *within a month of ECG (data available from 23,799 patients), **albumin-to-creatinine ratio >30mg/g (data available from 7,218 patients), ***within a month of ECG. AUC=area under the receiver operating characteristics curve, CI=Confidence interval, CKD=chronic kidney disease, eGFR=estimated glomerular filtration rate.

Proportion of available negative patients	CKD prevalence	AUC (95% CI)
100%	6.0%	0.770 (0.764-0.776)
50%	11.4%	0.769 (0.763-0.774)
25%	20.5%	0.769 (0.763-0.774)
10%	39.1%	0.764 (0.758-0.770)

Supplementary Table 4. Performance of different ECG-based deep learning models to detect any stage CKD. Models were trained with varying levels of CKD prevalence to illustrate the effect of CKD prevalence in the training set on the model performance. AUC=area under the receiver operating characteristics curve, CI=Confidence interval, CKD=chronic kidney disease.

Electrocardiographic characteristic	Overall	No CKD	Mild CKD	Moderate CKD	ESRD	P value
Heart rate, bpm	81.1 ±21.8	80.5 ±22.1	85.7 ±21.2	82.8 ±21.3	80.9 ±19.2	<0.001
PR interval, ms	166.0 ±37.7	164.5 ±36.9	165.8 ±36.8	172.5 ±43.2	168.4 ±34.6	<0.001
P wave duration, ms	52.2 ±9.6	52.1 ±9.2	52.1 ±11.2	51.9 ±11.1	53.6 ±9.2	<0.001
QRS duration, ms	101.0 ±29.0	98.7 ±27.2	112.6 ±36.3	111.8 ±35.6	98.9 ±24.7	<0.001
QTc interval, ms	457.6 ±66.7	452.6 ±70.3	477.4 ±60.2	474.5 ±55.2	468.3 ±43.7	<0.001
P-wave axis	50.2 ±27.5	50.3 ±27.0	50.6 ±26.6	50.2 ±29.9	48.9 ±26.2	<0.001
R-wave axis	23.6 ±60.2	23.2 ±51.1	38.0 ±85.1	24.6 ±75.1	22.1 ±57.2	<0.001
T-wave axis	57.5 ±57.6	54.1 ±54.9	61.1 ±64.4	67.6 ±66.3	67.0 ±57.3	<0.001

Supplementary Table 5. Electrocardiographic characteristics according to chronic kidney disease (CKD) stage in the training dataset. Data is presented as mean±standard deviation. ESRD=End-stage renal disease.

Model	AUC (95% CI)	Sensitivity (95% CI)	Specificity (95% CI)	PPV (95% CI)	NPV (95% CI)
**12-lead ECG models**					
Any stage CKD	0.709 (0.708-0.710)	0.573 (0.571-0.575)	0.720 (0.719-0.721)	0.276 (0.275-0.278)	0.900 (0.900-0.901)
Mild CKD	0.679 (0.675-0.682)	0.525 (0.519-0.530)	0.720 (0.719-0.721)	0.047 (0.046-0.048)	0.983 (0.983-0.983)
Moderate-severe CKD	0.714 (0.713-0.716)	0.585 (0.582-0.588)	0.720 (0.719-0.720)	0.141 (0.140-0.143)	0.956 (0.956-0.957)
ESRD	0.767 (0.764-0.769)	0.665 (0.660-0.670)	0.720 (0.719-0.721)	0.083 (0.082-0.084)	0.983 (0.982-0.983)
**High Risk Subgroup analyses for any stage CKD**					
Diabetic patients	0.699 (0.697-0.702)	0.620 (0.615-0.625)	0.670 (0.665-0.674)	0.711 (0.708-0.716)	0.573 (0.568-0.577)
Hypertensive patients	0.712 (0.710-0.714)	0.555 (0.552-0.558)	0.741 (0.740-0.742)	0.378 (0.375-0.380)	0.855 (0.854-0.856)
Age > 60 years	0.660 (0.658-0.661)	0.582 (0.579-0.584)	0.648 (0.647-0.649)	0.349 (0.347-0.350)	0.827 (0.826-0.828)
Male	0.719 (0.718-0.721)	0.593 (0.590-0.596)	0.721 (0.720-0.723)	0.387 (0.384-0.389)	0.857 (0.856-0.858)
Female	0.728 (0.726-0.730)	0.537 (0.533-0.541)	0.769 (0.768-0.770)	0.279 (0.276-0.281)	0.909 (0.908-0.910)
**Screening cohort (age < 60)**					
Any stage CKD	0.784 (0.782-0.786)	0.552 (0.548-0.556)	0.843 (0.843-0.845)	0.326 (0.323-0.328)	0.932 (0.931-0.933)
Mild CKD	0.763 (0.757-0.767)	0.524 (0.516-0.536)	0.843 (0.843-0.844)	0.063 (0.061-0.065)	0.989 (0.989-0.989)
Moderate-severe CKD	0.780 (0.777-0.782)	0.548 (0.543-0.556)	0.843 (0.843-0.844)	0.128 (0.126-0.130)	0.978 (0.978-0.979)
ESRD	0.841 (0.839-0.844)	0.649 (0.644-0.656)	0.843 (0.843-0.845)	0.155 (0.153-0.158)	0.982 (0.982-0.982)

Supplementary Table 6. Performance of the 12-lead ECG-based deep learning algorithm in the external dataset. AUC=area under the receiver operating characteristics curve, CI=Confidence interval, CKD=Chronic kidney disease, ESRD=End-stage renal disease. PPV=positive predictive value. NPV=negative predictive value.

Model	AUC (95% CI)	Sensitivity (95% CI)	Specificity (95% CI)	PPV (95% CI)	NPV (95% CI)
**1-lead ECG models**					
**Any stage CKD**	0.701 (0.700-0.702)	0.660 (0.658-0.662)	0.635 (0.634-0.635)	0.252 (0.251-0.253)	0.909 (0.908-0.910)
**Mild CKD**	0.671 (0.668-0.674)	0.617 (0.612-0.621)	0.635 (0.634-0.636)	0.043 (0.042-0.043)	0.984 (0.984-0.985)
**Moderate-severe CKD**	0.694 (0.692-0.695)	0.649 (0.645-0.652)	0.635 (0.634-0.636)	0.123 (0.122-0.124)	0.958 (0.958-0.959)
**ESRD**	0.780 (0.778-0.782)	0.784 (0.780-0.788)	0.635 (0.634-0.636)	0.075 (0.075-0.076)	0.987 (0.987-0.987)
**High Risk Subgroup analyses for any stage CKD**					
**Diabetic patients**	0.678 (0.674-0.681)	0.710 (0.706-0.713)	0.544 (0.539-0.549)	0.671 (0.667-0.674)	0.588 (0.584-0.593)
**Hypertensive patients**	0.697 (0.696-0.699)	0.627 (0.623-0.630)	0.661 (0.659-0.662)	0.343 (0.340-0.346)	0.862 (0.861-0.863)
**Age > 60 years**	0.657 (0.655-0.658)	0.646 (0.643-0.649)	0.583 (0.582-0.585)	0.335 (0.332-0.336)	0.836 (0.834-0.837)
**Male**	0.722 (0.720-0.723)	0.667 (0.665-0.669)	0.661 (0.659-0.662)	0.368 (0.366-0.370)	0.870 (0.869-0.871)
**Female**	0.706 (0.704-0.708)	0.648 (0.644-0.652)	0.650 (0.648-0.651)	0.236 (0.234-0.237)	0.917 (0.916-0.918)
**Screening cohort (age < 60)**					
**Any stage CKD**	0.777 (0.775-0.779)	0.694 (0.690-0.697)	0.728 (0.726-0.729)	0.259 (0.257-0.261)	0.946 (0.945-0.946)
**Mild CKD**	0.752 (0.748-0.757)	0.657 (0.647-0.666)	0.728 (0.726-0.729)	0.046 (0.045-0.047)	0.991 (0.990-0.991)
**Moderate-severe CKD**	0.764 (0.761-0.768)	0.674 (0.668-0.679)	0.728 (0.726-0.729)	0.094 (0.092-0.095)	0.982 (0.981-0.982)
**ESRD**	0.837 (0.834-0.839)	0.799 (0.794-0.804)	0.728 (0.727-0.729)	0.115 (0.114-0.117)	0.988 (0.988-0.988)

Supplementary Table 7. Performance of the 1-lead ECG-based deep learning algorithm in the external dataset. AUC=area under the receiver operating characteristics curve, CI=Confidence interval, CKD=Chronic kidney disease, ESRD=End-stage renal disease. PPV=positive predictive value. NPV=negative predictive value.

	No CKD<br><br>n=20,770	Mild CKD<br>(Stage 1-2)<br>n=293	Moderate CKD<br>(Stage 3-5)<br>n=1843	ESRD<br><br>n=893
eGFR (by CKD-EPI formula)	72.1% >60 without point estimate	40.77 ±13.04	30.48 ±15.02	10.47 ±9.50

Supplementary Table 8. Distribution of estimated glomerular filtration rate (eGFR) values according to chronic kidney disease (CKD) stage in the training dataset. Values are presented as mean±standard deviation. eGFR values are taken within a month of ECG. ESRD=End-stage renal disease.

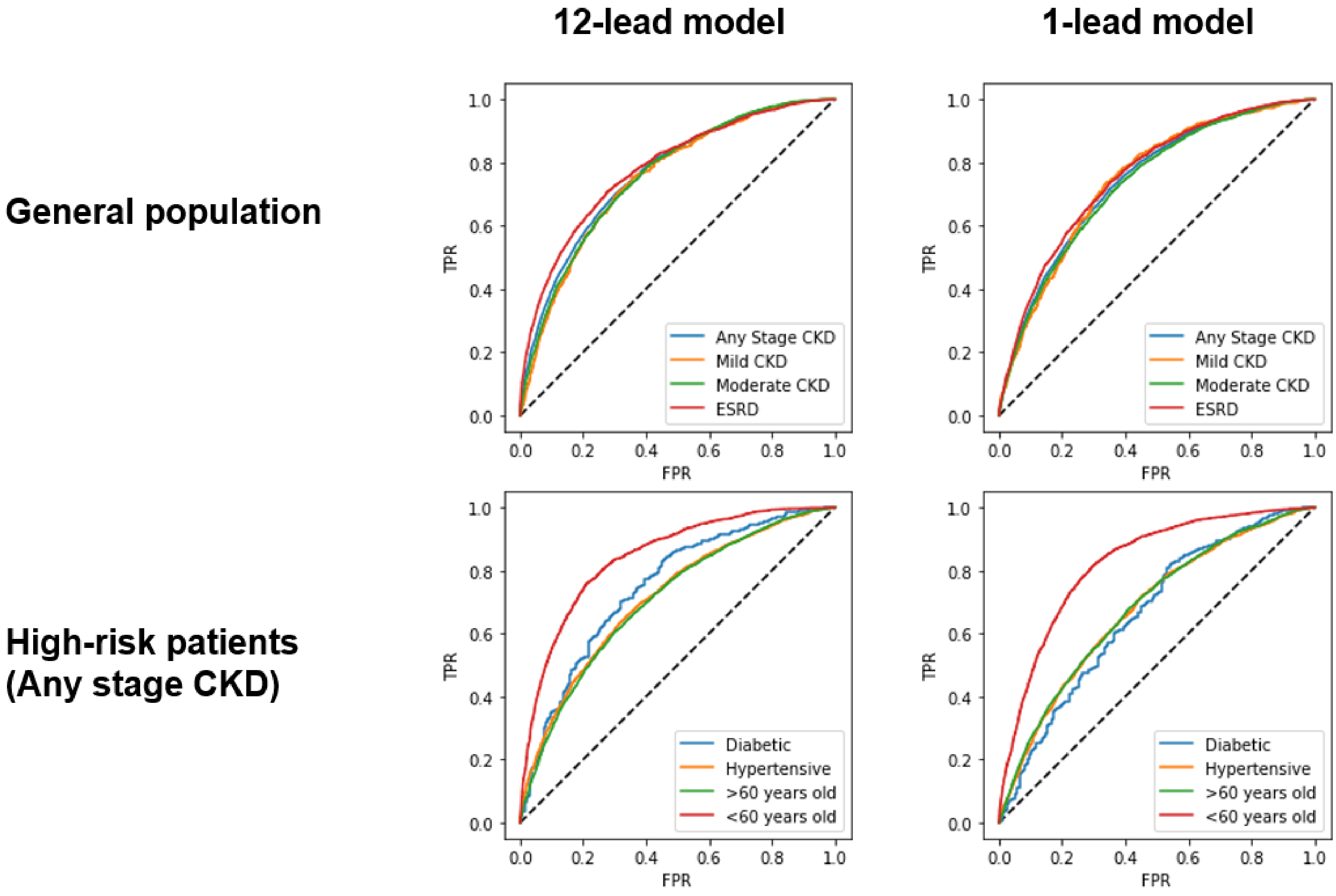

Supplementary Figure 1. Model performance in the internal dataset. CKD=Chronic kidney disease. ESRD=End-stage renal diseas

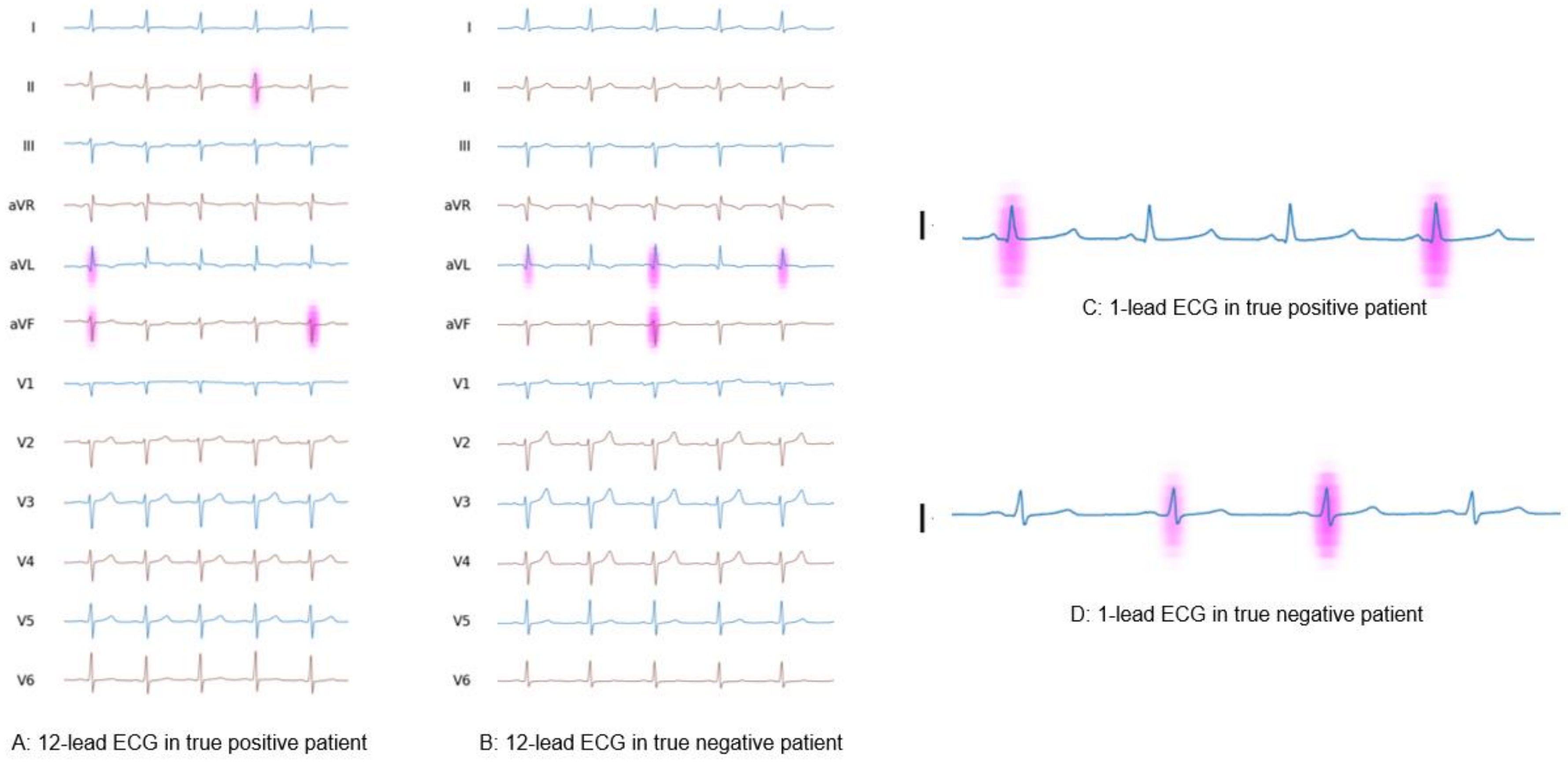

Supplementary Figure 2. The Linear Interpretable Model-Agnostic Explanations (LIME) map of 12-lead and 1-lead ECGs highlights features that were important for deep learning model in chronic kidney disease detection. Important ECG features are highlighted with greater color intensity. In both true positive (A and C) and true negative patients (B and D) LIME highlighted QRS complexes and PR intervals.

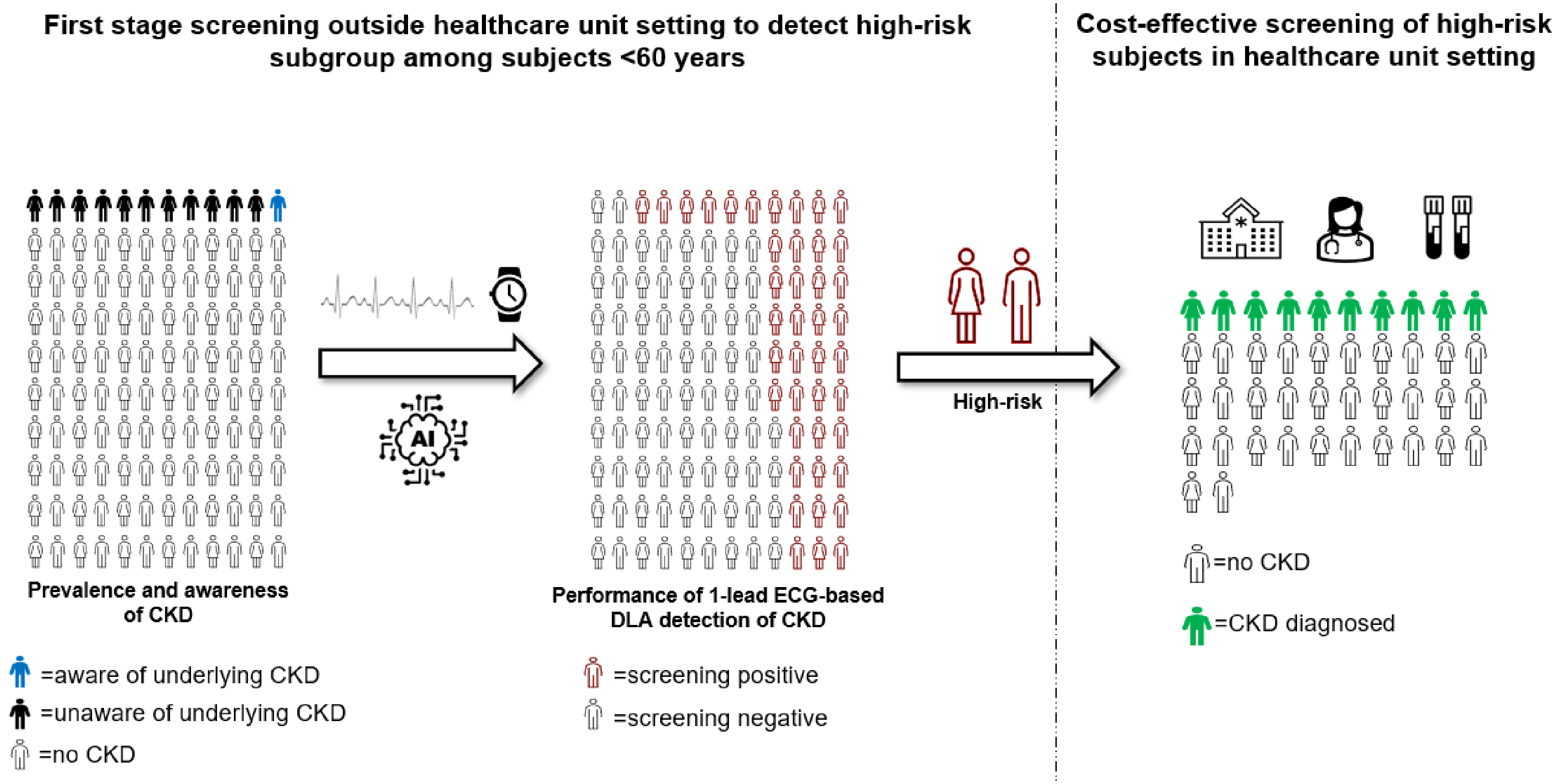

Supplementary Figure 3. Schematic illustration of utilizing ECG-based deep learning model to detect a high-risk subgroup of subjects under 60 years of age for chronic kidney disease (CKD) screening. DLA=Deep learning algorithm.

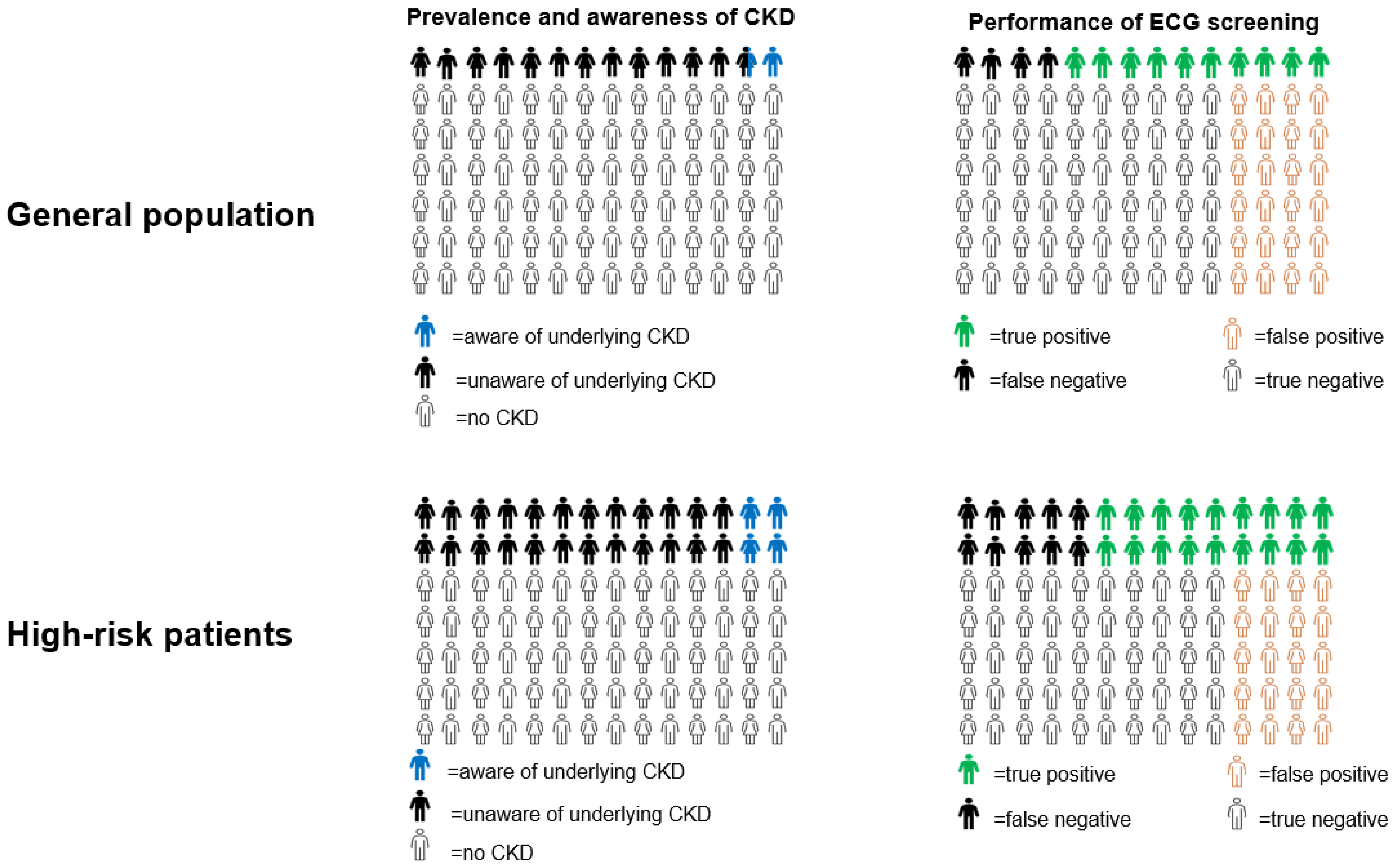

Supplementary Figure 4. Illustration of chronic kidney disease (CKD) prevalence and awareness in comparison to model performance.

Supplementary References

1. Sandler M, Howard A, Zhu M, Zhmoginov A and Chen L-C. MobileNetV2: Inverted Residuals and Linear Bottlenecks. arXiv [csCV]. 2018.

2. Tan M and Le QV. EfficientNet: Rethinking Model Scaling for Convolutional Neural Networks. arXiv [csLG]. 2019.

3. Davis C, Tait G, Carroll J, Wijeysundera DN and Beattie WS. The Revised Cardiac Risk Index in the new millennium: a single-centre prospective cohort re-evaluation of the original variables in 9,519 consecutive elective surgical patients. Can J Anaesth. 2013;60:855-863.

4. Ford MK, Beattie WS and Wijeysundera DN. Systematic review: prediction of perioperative cardiac complications and mortality by the revised cardiac risk index. Ann Intern Med. 2010;152:26-35.

5. Gupta PK, Gupta H, Sundaram A, Kaushik M, Fang X, Miller WJ, Esterbrooks DJ, Hunter CB, Pipinos II, Johanning JM, Lynch TG, Forse RA, Mohiuddin SM and Mooss AN. Development and validation of a risk calculator for prediction of cardiac risk after surgery. Circulation. 2011;124:381-387.

6. https://arxiv.org/abs/2205.03242

7. https://github.com/echonet/PreOpNet
