# Supplementary material for: Deep learning-based electrocardiographic screening for chronic kidney disease
Source: Commun Med (Lond). 2023 May 26;3:73. doi: 10.1038/s43856-023-00278-w (PMC10220039; doi:10.1038/s43856-023-00278-w)
Supplement: Supplementary file 2 — Reporting Summary [file 43856_2023_278_MOESM2_ESM.pdf]

nature portfolio

Corresponding author(s): David Ouyang, MD
Cedars-Sinai Medical Center
127 S. San Vicente Blvd.
AHSP Pavilion Suite A3100
Los Angeles, CA 90048
David.Ouyang@cshs.org

Last updated by author(s): Feb 14, 2023

Reporting Summary

Nature Portfolio wishes to improve the reproducibility of the work that we publish. This form provides structure for consistency and transparency in reporting. For further information on Nature Portfolio policies, see our Editorial Policies and the Editorial Policy Checklist.

Statistics

For all statistical analyses, confirm that the following items are present in the figure legend, table legend, main text, or Methods section.

n/a	Confirmed	
☐	☒	The exact sample size (*n*) for each experimental group/condition, given as a discrete number and unit of measurement
☐	☒	A statement on whether measurements were taken from distinct samples or whether the same sample was measured repeatedly
☐	☒	The statistical test(s) used AND whether they are one- or two-sided<br>*Only common tests should be described solely by name; describe more complex techniques in the Methods section.*
☐	☒	A description of all covariates tested
☒	☐	A description of any assumptions or corrections, such as tests of normality and adjustment for multiple comparisons
☐	☒	A full description of the statistical parameters including central tendency (e.g. means) or other basic estimates (e.g. regression coefficient) AND variation (e.g. standard deviation) or associated estimates of uncertainty (e.g. confidence intervals)
☒	☐	For null hypothesis testing, the test statistic (e.g. *F*, *t*, *r*) with confidence intervals, effect sizes, degrees of freedom and *P* value noted<br>*Give P values as exact values whenever suitable.*
☒	☐	For Bayesian analysis, information on the choice of priors and Markov chain Monte Carlo settings
☒	☐	For hierarchical and complex designs, identification of the appropriate level for tests and full reporting of outcomes
☒	☐	Estimates of effect sizes (e.g. Cohen's *d*, Pearson's *r*), indicating how they were calculated

Our web collection on statistics for biologists contains articles on many of the points above.

Software and code

Policy information about availability of computer code

Data collection	No software was used
Data analysis	Statistical analysis was performed in R and Python, for which code is available at doi:10.5281/zenodo.7713558.

For manuscripts utilizing custom algorithms or software that are central to the research but not yet described in published literature, software must be made available to editors and reviewers. We strongly encourage code deposition in a community repository (e.g. GitHub). See the Nature Portfolio guidelines for submitting code & software for further information.

Data

Policy information about availability of data

All manuscripts must include a data availability statement. This statement should provide the following information, where applicable:

- Accession codes, unique identifiers, or web links for publicly available datasets
- A description of any restrictions on data availability
- For clinical datasets or third party data, please ensure that the statement adheres to our policy

All code and analytical methods applied for the deep learning algorithm are included in this published article, supplementary files, and at https://github.com/ecg-net/CKDscreening. The patient data is not publicly available due to potentially identifiable nature of the associated data.

Human research participants

Policy information about studies involving human research participants and Sex and Gender in Research.

Reporting on sex and gender	We have performed secondary analyses separately for male and female
Population characteristics	We have provided demographic and clinical characteristics of our study subjects in Table 1.
Recruitment	Study subjects and associated clinical data were identified from Cedars-Sinai Medical Center (between 2005-2019) and Stanford Healthcare (from 8/2005 to 6/2018) electronic health records
Ethics oversight	The institutional review boards of Cedars-Sinai Medical Center and Stanford Healthcare approved the study protocol

Note that full information on the approval of the study protocol must also be provided in the manuscript.

Field-specific reporting

Please select the one below that is the best fit for your research. If you are not sure, read the appropriate sections before making your selection.

☒ Life sciences ☐ Behavioural & social sciences ☐ Ecological, evolutionary & environmental sciences

For a reference copy of the document with all sections, see nature.com/documents/nr-reporting-summary-flat.pdf

Life sciences study design

All studies must disclose on these points even when the disclosure is negative.

Sample size	We retrospectively identified 54,582 ECGs among 7,947 patients between 2005-2019 which were linked to a diagnosis of CKD within a 1-year window at Cedars-Sinai Medical Center. We also identified 193,073 ECGs among 103,814 patients between 2008-2019 with no CKD diagnoses at any point, which were used as matched negative controls. Similarly, we identified 896,620 ECGs among 312,145 patients at Stanford Healthcare from 8/2005 to 6/2018, which were used for external validation.
Data exclusions	We excluded subjects without ECG or if their ECG was linked to a diagnosis of CKD more than 1-year apart.
Replication	The model and results were externally validated using an external cohort from another healthcare system (Stanford Healthcare)
Randomization	The study population from Cedars-Sinai Medical Center was randomly split 8:1:1 into training, validation, and test cohorts by patient such that the multiple ECGs from the same patient were limited to one cohort
Blinding	Blinding was not relevant to train, test, and externally validate the deep learning model

Reporting for specific materials, systems and methods

We require information from authors about some types of materials, experimental systems and methods used in many studies. Here, indicate whether each material, system or method listed is relevant to your study. If you are not sure if a list item applies to your research, read the appropriate section before selecting a response.

Materials & experimental systems

n/a	Involved in the study	
☒	☐	Antibodies
☒	☐	Eukaryotic cell lines
☒	☐	Palaeontology and archaeology
☒	☐	Animals and other organisms
☐	☒	Clinical data
☒	☐	Dual use research of concern

Methods

n/a	Involved in the study	
☒	☐	ChIP-seq
☒	☐	Flow cytometry
☒	☐	MRI-based neuroimaging

Clinical data

Policy information about clinical studies

All manuscripts should comply with the ICMJE guidelines for publication of clinical research and a completed CONSORT checklist must be included with all submissions.

Clinical trial registration	This is not a clinical trial
Study protocol	This is not a clinical trial
Data collection	Study subjects and associated clinical data were retrospectively collected from electronic health records: 54,582 ECGs among 7,947 patients between 2005-2019 which were linked to a diagnosis of CKD within a 1-year window at Cedars-Sinai Medical Center. We also identified 193,073 ECGs among 103,814 patients between 2008-2019 with no CKD diagnoses at any point, which were used as matched negative controls. Similarly, we identified 896,620 ECGs among 312,145 patients at Stanford Healthcare from 8/2005 to 6/2018, which were used for external validation.
Outcomes	The primary outcome was any-stage CKD within 1 year of ECG. Secondary sensitivity analyses were limited to procedures performed in different CKD stages and in patients with diabetes, hypertension, male, and age greater or lower than 60 years old.
